# Tau pathology in the medial temporal lobe of athletes with chronic traumatic encephalopathy: a chronic effects of neurotrauma consortium study

**DOI:** 10.1186/s40478-019-0861-9

**Published:** 2019-12-12

**Authors:** Christy M. Kelley, Sylvia E. Perez, Elliott J. Mufson

**Affiliations:** Department of Neurobiology and Neurology, Barrow Neurological Institute, 350 West Thomas Rd, Phoenix, AZ 85013 USA

**Keywords:** Chronic traumatic encephalopathy, Tau, Athletes, Medial temporal lobe, Hippocampus, Repetitive traumatic brain injury

## Abstract

Chronic traumatic encephalopathy (CTE) is a progressive neurodegenerative condition associated with repetitive traumatic brain injury (rTBI) seen in contact-sport athletes and military personnel. The medial temporal lobe (MTL; i.e., hippocampus, subiculum, and entorhinal and perirhinal cortices) memory circuit displays tau lesions during the pathological progression of CTE. We examined MTL tissue obtained from 40 male Caucasian and African American athletes who received a postmortem CTE neuropathological diagnosis defined as stage II, III, or IV. Sections were immunolabeled using an early (AT8) or a late (TauC3) marker for pathological tau and for amyloid beta (Aβ) species (6E10, Aβ_1–42_ and thioflavin S). Stereological analysis revealed that stage III had significantly less AT8-positive neurons and dystrophic neurites than stage IV in all MTL regions except hippocampal subfield CA3, whereas significantly more AT8-positive neurons, dystrophic neurites, and neurite clusters were found in the perirhinal cortex, entorhinal cortex, hippocampal CA1, and subiculum of CTE stage III compared with stage II. TauC3-positive pathology was significantly higher in the perirhinal and subicular cortex of stage IV compared to stage III and the perirhinal cortex of stage III compared to stage II. AT8-positive neurite clusters were observed in stages III and IV, but virtually absent in stage II. When observed, Aβ pathology appeared as amyloid precursor protein (APP)/Aβ (6E10)-positive diffuse plaques independent of region. Thioflavine S labeling, did not reveal evidence for fibril or neuritic pathology associated with plaques, confirming a diffuse, non-cored plaque phenotype in CTE. Total number of AT8-positive profiles correlated with age at death, age at symptom onset, and time from retirement to death. There was no association between AT8-positive tau pathology and age sport began, years played, or retirement age, and no difference between CTE stage and the highest level of sport played. In summary, our findings demonstrate different tau profiles in the MTL across CTE stages, proffering CA3 tau pathology and MTL dystrophic neurite clusters as possible markers for the transition between early (II) and late (III/IV) stages, while highlighting CTE as a progressive noncommunicative tauopathy.

## Introduction

Repetitive traumatic brain injury (rTBI) plays a key role in the development of chronic traumatic encephalopathy (CTE), a progressive neurodegenerative disorder characterized by the widespread deposition of hyperphosphorylated tau (p-tau) within the brain [21–25, 31–33, 35, 41, 62, 63, 77, 78, 97]. Military personnel and athletes in contact sports (e.g., boxing, American football, and hockey) are exposed to rTBI at greater rates than non-athletes or civilians [62, 63, 65, 72, 77–79] and frequently develop age-related clinical signs of dementia including cognitive impairment, memory loss and neuropsychiatric sequelae (e.g., depression, impulsivity, and aggression) [66, 96]. Currently, CTE diagnosis is made postmortem based on neuropathology that structurally and functionally underlies the clinical presentation [1, 4, 28, 55, 62, 107].

CTE neuropathology is characterized by p-tau positive glia, intraneuronal neurofibrillary tangles (NFTs) and small punctate aggregates, which appear early in the frontal cortex and later in the medial temporal lobe (MTL), primarily in association with small cerebral vessels in the depths of sulci [6, 33, 65]. The MTL memory circuit is also a primary site of tau pathology in the aged [72] and Alzheimer’s disease (AD) brain [15]. Clinicopathological studies have shown that NFTs are an excellent correlate of cognitive impairment in AD and other tauopathies, indicating a strong association between affected brain structure and functional impairment [44, 103]. In contrast to AD Braak staging where NFT tau pathology begins in the MTL, for CTE the disease-associated MTL tau pathology is not observed until later stages while the frontal cortex is affected early [15, 61, 65]. To our knowledge there have been no rigorous quantitative studies of p-tau lesions in the MTL of CTE brains [65, 72]. Interestingly, in contrast to extensive amyloid pathology seen in the MTL in AD [71], amyloid-beta (Aβ)-positive plaques are not a consistent finding in CTE, and it is unclear whether their presence represents a state distinct from aging without rTBI [2].

Here we used an unbiased counting procedure to quantify the types of tau pathology within the MTL of CTE cases that received a postmortem neuropathological designation of stage II, III, or IV according to the McKee schema [61]. The data shed new light on the distribution and progression of p-tau pathology within this highly intraconnective memory network. Since past reports have highlighted CTE stage II as having minimal tau pathology in the MTL, we chose an early marker for p-tau lesions, AT8. Prior reports have established that the AT8 antibody directed against tau phosphorylated at serine 202 and threonine 205 (pS202/T205) is an effective tool for the post mortem staging of CTE [61]. Additionally, we analyzed a subgroup of subjects using the late stage tau marker, TauC3, which recognizes tau truncated at aspartic acid 421 (D421) [52, 75]. Lastly, we determined the extent of amyloid-like pathology in the MTL using antibody immunohistochemistry and thioflavine S histochemistry. The overall aim of this study was to characterize p-tau structures in MTL tissue obtained at autopsy from former contact-sport athletes, to provide greater insight into the spectrum of CTE-associated pathology, which will lay a foundation for future prospective longitudinal studies and therapeutic targeting of select tau epitopes at different CTE stages.

## Materials and methods

### Subjects

**Fig. 1:**
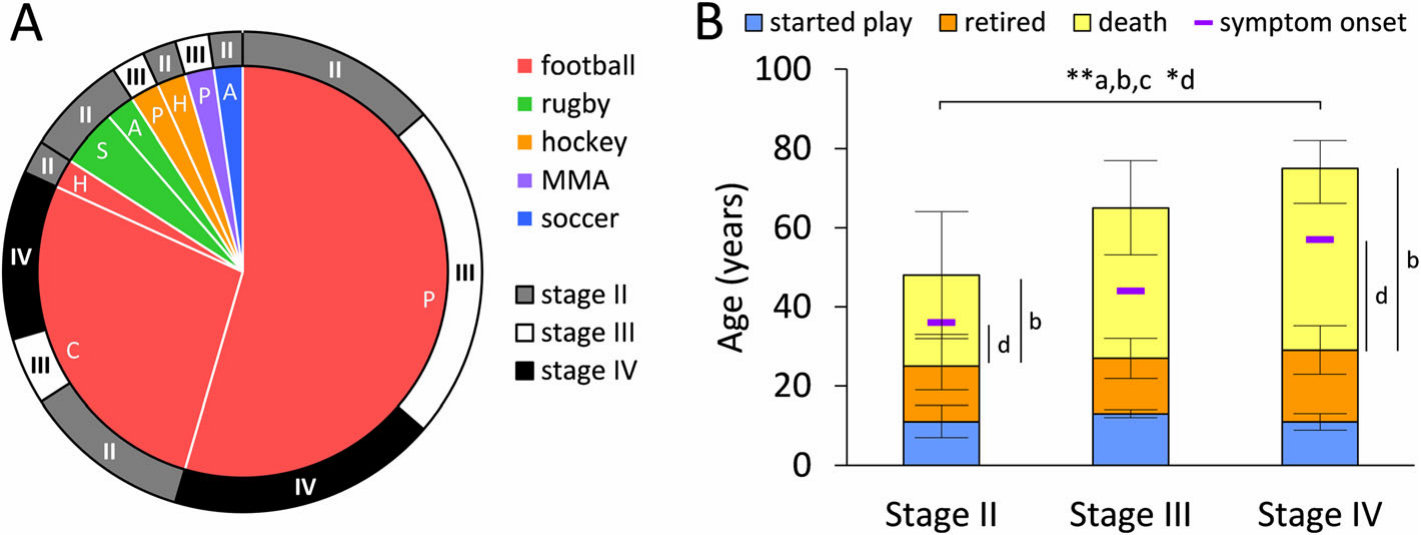
Graphs show a summary of sports played by CTE stage in a circular pie and doughnut plot, and the average timeline of athlete’s career across stage in a bar graph. **A** Sport and maximum level at which it was played is shown in the inner pie graph, while the distribution across stages is displayed in the outer doughnut plot for the 40 athletes. Some athletes reported playing multiple sports, but there is only one level for each sport. Inner graph shows a pie plot with colors denoting sport (red, football, *n* = 37; green, rugby, *n* = 3; orange, hockey, *n* = 2; purple, mixed martial arts (MMA), *n* = 1; blue, soccer, *n* = 1) and letters denoting the maximum level at which the athlete(s) played that sport (P, professional, *n* = 26; C, college, *n* = 12; S, semi-professional, *n* = 2; A, amateur, *n* = 2; and H, high school, *n* = 2). Outer graph shows a doughnut plot with the distribution across CTE stages for the corresponding pie. **B** Stacked bar graph shows average age (mean with standard deviation) athlete started playing (top line of blue box), years played (orange) and retirement age (top line of orange box), age at death (top line of yellow box) and age of symptom onset (purple horizontal marker) per CTE stage. There were significant differences between stages II and IV in age at death (a), years from retirement to death (b), age at symptom onset (c), and years from retirement to symptom onset (d). No differences were found between age athlete started playing contact sports, the number of years played, the age at retirement, or time from symptom onset to death across CTE stage. * *p* < 0.025, ** *p* < 0.005 KWDB

Tissue sections containing the MTL were obtained from the brains of 40 male contact sport athletes (American football, hockey, rugby, soccer, and mixed martial arts) (Fig. 1A). Each case underwent a post mortem tau-based CTE staging examination [61, 94]. Institutional review board approval for brain donation was obtained through the Boston University Alzheimer’s Disease Center CTE program and the Bedford Veterans’ Affairs hospital. Institutional review board approval for post-mortem review of clinical records, family member interviews and neuropathological analysis was obtained through Boston University School of Medicine. Consent for tissue donations was attained from the subject or a family member and is part of a larger brain bank collaboration (VA-BU-CLF Brain Bank, http://www.bu.edu/cte/our-research/brain-bank/).

### Symptom assessment

Evaluation of the presence and timeline of symptoms (cognitive, emotional, and personality alterations) was performed as previously reported [67]. Briefly, a retrospective evaluation was conducted to acquire subject information including symptom onset, military veteran status, and athletic career timeline. Evaluation includes a combination of online surveys and telephone interviews between research personnel and either family members or close friends of the deceased athlete. Researchers performing the evaluations were blinded to CTE pathological stage. Information detailing the number of head injuries sustained, prevalence of rTBI between the different contact sports and episodes of impact-associated loss of consciousness was not available.

### CTE staging

CTE staging was based on a National Institute for Neurological Disorders and Stroke (NINDS) and National Institute of Biomedical Imaging and Bioengineering (NIBIB) consortium consensus criteria, which includes identification of perivascular p-tau accumulation within neuronal perikarya and processes, and astrocytes, appearing in an intermittent discontinuous distribution concentrated within the depths of sulci [61]. CTE staging uses four stereotypic distribution patterns of AT8-positive p-tau pathology throughout the cortex and brain stem [61, 64, 65]. Briefly, in stage I, histological evaluation reveals sparse and isolated occurrences of perivascular p-tau-immunoreactive neurons, neuropil threads, and astrocytes mostly in the depths of cerebral sulci of the superior, dorsolateral, lateral, and inferior frontal cortices, with no p-tau pathology in the MTL. Stage II displays multiple p-tau foci concentrated in the depths of cortices for frontal (superior, dorsolateral, lateral, and inferior), temporal (anterior, inferior, and lateral), parietal (inferior and superior), and insula cortex and septal region. In contrast to stage I, in stage II p-tau lesions are found in the superficial layers of the cerebral cortex extending to the gyral crest as well as subcortical and brainstem nuclei. Stage III exhibits intraneuronal p-tau NFTs found diffusely in the frontal, temporal, parietal, and insular cortices concentrated around small vessels and within the depths of sulci. Additionally, in stage III, the hippocampus, entorhinal cortex, amygdala, nucleus basalis of Meynert, and locus coeruleus show extensive p-tau pathology. Stage IV displays widespread NFTs in neurons and astrocytes throughout frontal, parietal and MTL cortical regions, as well as the diencephalon and brainstem nuclei (for details see [61, 64–66]). Importantly, this staging schema is dissimilar from Braak staging of AD and presents a necessarily distinct diagnostic criterion [15, 61].

### Immunohistochemistry

Tissue blocks containing the more rostral aspect of the MTL extending from the pes hippocampi to the rostral substantia nigra at the level of the caudal external globus pallidus, entorhinal and subicular cortex were paraffin embedded, sectioned at 7 μm and mounted on glass slides. Sections were deparaffinized in xylenes, rehydrated in decreasing grades of ethanol (100, 95, 70, and 50%), and rinsed in distilled water (dH_2_O). Following citrate buffer antigen retrieval (2.1 g citric acid per 1 L dH_2_O; for AT8 and TauC3) or formic acid antigen retrieval (88%; for 6E10 and Aβ_1–42_), slides were rinsed in phosphatase buffer (PB) and tris-buffered saline (TBS) [47, 72]. Briefly, slides were incubated in sodium meta periodate (2.139 g: 1 L TBS 1X), washed in TBS/Triton X-100 (0.25%), soaked in TBS/Triton X-100 with 3% goat serum, and incubated overnight at room temperature (RT) in TBS/Triton X-100 with 1% goat serum containing a monoclonal antibody against AT8 (1:500; Fisher Scientific, Hampton, NH, USA), a marker for paired helical filament tau phosphorylated at S202/T205; a monoclonal antibody against TauC3 (1:50), a marker for pathological tau cleaved at D421 (Invitrogen, Carlsbad, CA, USA); 6E10 (1:1000), a marker for amyloid precursor protein (APP) and the cleavage products amyloid beta (Aβ) and APP intracellular domain (AICD) (Covance, Princeton, NJ, USA); or anti-Aβ_1–42_ (1:100), a marker for soluble and fibrillar Aβ_1–42_ that does not cross-react with Aβ_1–40_, APP, or AICD (Millipore, Burlington, MA, USA) [42]. Following antibody incubation, slides were washed in TBS 1X with 1% goat serum, incubated for 1 h in biotinylated goat anti-mouse IgG (1:200), washed in TBS, incubated for 1 h in avidin-biotin complex (ABC) solution (Vectastain, Vector Laboratories, Burlingame, CA, USA), washed in sodium imidazole acetate buffer and visualized in a solution containing either diaminobenzidine (DAB, Sigma-Aldrich, St. Louis, MO, USA) and nickel(II) ammonium sulfate (AT8, TauC3) or DAB alone (6E10, Aβ_1–42_). Slides were rinsed, dehydrated in increasing grades of ethanol followed by xylenes and coverslipped with DPX mounting medium (Electron Microscopy Sciences, Hatfield, PA, USA). Negative controls consisted of primary or secondary antibody deletion followed by the protocol described above. Both controls were negative for immunolabeling, cross-reactivity, or artifacts. Positive controls for antibody reactivity were performed using AD tissue.

### Thioflavine S histochemistry

Slides from a subset of subjects were labeled with thioflavine S to verify whether plaques contained amyloidogenic fibrils. Sections processed for thioflavine S were defatted in 1:1 chloroform and 100% ethanol for 2 h, rehydrated as above, and stained with 0.5% aqueous thioflavine S (Sigma-Aldrich) for 20 min at RT, followed by differentiation in 80% ethanol. After several dH_2_O rinses, slides were coverslipped with an aqueous mounting medium (Gel-Mount; Biomeda, Foster City, CA, USA). Thioflaving S was visualize with the aid of a Nikon Optiphot-2 fluorescence microscope.

### Region of interest

**Fig. 2:**
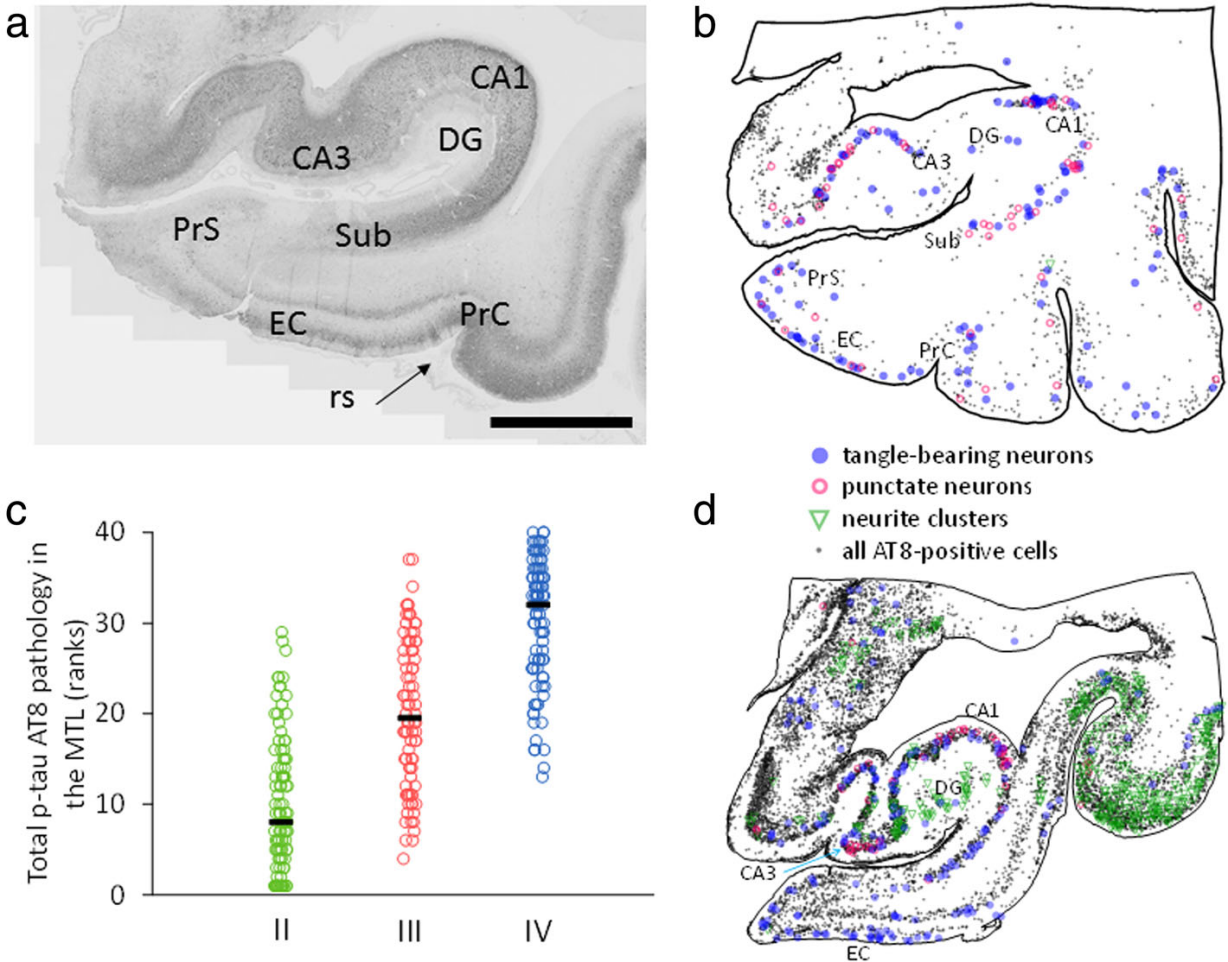
Photomicrograph, topographical maps and scatterplot of AT8 immunolabeling in the medial temporal lobe (MTL). **a** Low magnification image showing areas of the MTL evaluated for AT8 immunoreactivity. Abbreviations: PrC, perirhinal cortex; EC, entorhinal cortex; DG, dentate gyrus; CA3 and CA1, hippocampal subfields; Sub, subiculum; PrS, pre−/parasubiculum (arrow indicates rhinal sulcus). Note the lamina distribution of AT8-positive labeling in CA1, EC layers V-VI and II, and PrC. Scale bar = 5 mm. **b** and **d** Representative topographic maps show distribution of AT8 immunopositive tangle-bearing neurons (blue solid circle), neurons displaying puncta (pink open circle), neurite clusters (green triangle), glia and small local interneurons (gray dots) in CTE stage III (**b**) and stage IV (**d**). Note the increase in the extent and density of the various tau pathologies in stage IV compared to stage III. **c** Staggered scatterplot demonstrates pathology overlap across CTE stages. While there is large overlap, pathology clusters at the low end in stage II (green) and at the high end in stage IV (blue). Each data point represents a subject:region pairing

Tau pathology was quantitatively evaluated and the distribution charted across seven MTL regions including: hippocampal CA1 and CA3 subfields, dentate gyrus (DG), subiculum (Sub), pre−/parasubiculum (PrS), entorhinal cortex (EC) and perirhinal cortex (PrC) (Fig. 2a), using a Nikon Optiphot-2 light microscope controlled by Stereo Investigator software (MBF Bioscience, Williston, VT, USA) loaded on a Dell computer. Since the hippocampal sections analyzed were limited to its more rostral regions and angle of cut varied, a well-defined CA4 subfield was difficult to consistently discern. MTL regions were mapped at 100X magnification (10X lens) using the DG granule layer as a guide and topographical location defined according to the human brain atlas of Mai and colleagues [60] (serial plate numbers: 41–47, with position ranges: MNI [− 9.66]–[− 18.30], ICL [9.22]–[17.45], MCP [− 5.52]–[2.71]).

### Tau profile quantitation

**Fig. 3:**
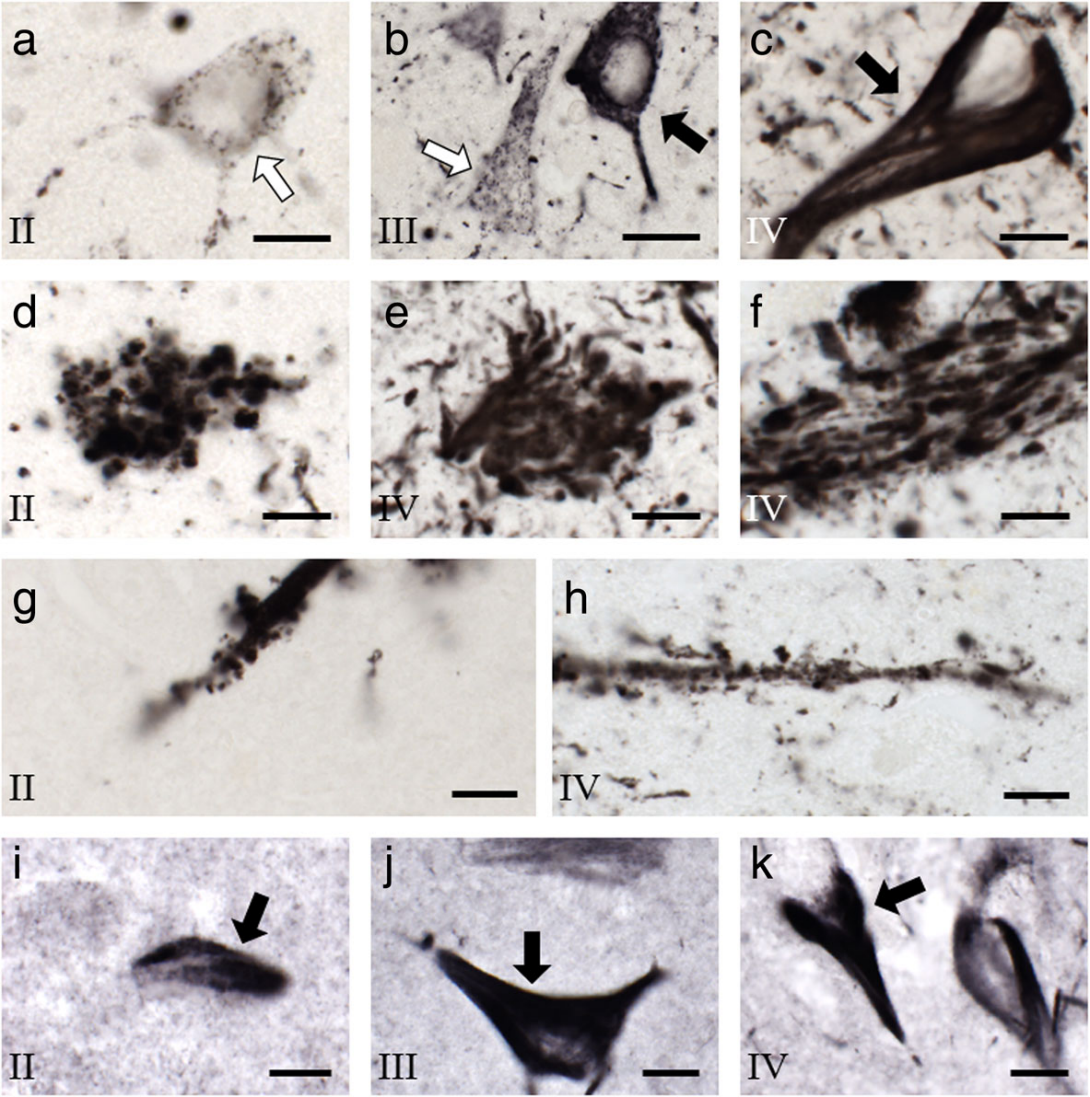
High-magnification photomicrographs of hippocampal sections immunolabeled for tau from representative CTE stage II, III, and IV contact-sport cases. **a**–**c** Photomicrographs of AT8-positive neurons show intense, confluent labeling (black arrows) or puncta (white arrows) in hippocampal CA1 of stage II (**a**), III (**b**), and IV (**c**). **d**–**f** Photomicrographs of AT8-positive neurite clusters within CA1 from stage II (**d**) and IV (**e**) and CA3 hippocampal subfields from stage IV (**f**). **g** and **h** Example of a dystrophic fiber showing thorny excrescences in CA3 of a stage II case (**g**), and a non-dystrophic fiber with thorny excrescences in CA3 of a stage IV case (**h**). Thorny excrescences are distinct from artifacts seen with DAB/Ni processing [13]. **i**–**k** TauC3-immunolabeled CA1 neurons in stage II (**i**), III (**j**), and IV (**k**). Note the confluent deep labeling across the perikarya of neurons (black arrows). Scale bars = 10 μm in all panels. Roman numerals represent CTE stage

AT8- and TauC3-positive structures (i.e., neuronal and non-neuronal) were counted at 600X magnification using a 60X oil-immersion lens (n.a. 1.40). Forty cases were used for AT8 analyses (Table 1) and a subsample of 15 cases for which we had extra tissue were immunolabeled for TauC3 (stage II *n* = 5, mean age at death 39.6 years; stage III *n* = 5, mean age at death 65.8 years; stage IV *n* = 5, mean age at death 73.0 years). For p-tau positive neurons, only those profiles displaying an axon or dendrites were counted [9]. Dystrophic neurites were operationally defined as having a width of ≥3 μm extending for ≥6 μm (e.g., Fig. 3g profile met criteria, as opposed to Fig. 3h). Stereological systematic sampling with a random start within a grid sized 313 μm × 313 μm coupled with an optical disector-like probe of 90 μm × 120 μm (use of x and y inclusion and exclusion lines with no height boundaries) provided an 11.0% sampling of each region (145 average number of sites per region per subject). The number of profiles was derived using the inverse of the sampling fraction (1/0.1102) for a multiplication factor, and final numbers indicate counts per 10,000,000 μm^2^ to avoid bias imposed by alternate cut angles or regional size differences across brains. Due to limits in human brain tissue availability, we were not able to process the entire length of the hippocampal formation and could not calculate a total regional estimate of profile number.

**Table 1:** Descriptive statistics of CTE groups

Stage	n	Race (AA:C)^a^	Military veteran (n)	Age at death^b^	Age started contact sports	Years played	Age retired	Time from retirement to death (years)
II	14	1:13	1	48 ± 16 (24–71)	11 ± 4 (5–18)	14 ± 6 (5–27)	26 ± 7 (18–42)	23 ± 17 (1–46)
III	13	7:6	0	65 ± 12 (41–80)	13 ± 1 (10–14)	14 ± 5 (6–25)	27 ± 5 (19–35)	38 ± 15 (11–57)
IV	13	2:11	2	74 ± 8 (62–86)	11 ± 2 (8–14)	18 ± 7 (8–30)	29 ± 6 (21–40)	46 ± 12 (29–64)

^a^*AA* African American, *C* Caucasian

^b^numbers presented as mean ± SD (range)

Size of AT8-positive neurons and neurite clusters was measured on a subset of subjects, selected prior to any analyses, for representation across groups based on subject demographics (stage II *n* = 8, stage III *n* = 6, stage IV *n* = 8; two subjects were excluded due to poor tissue quality) using a 60X oil-immersion lens and an eight-ray nucleator probe [39]. The nucleator probe involves placing a marker at the center of a neuron or neurite cluster, from which 8 rays at 45° intervals emanate and additional markers are placed at the locations where each ray bisects the periphery of the perikaryon or cluster in a single plane of focus (0.1 μm z-axis forgiveness). From this, an average radius is calculated and used to derive a cross-sectional area measurement using classic geometric principles with a shape factor assumption of ~ 3–4 [39]. Thickness of swollen, dystrophic neurites was measured using a line measurement tool in the Stereo Investigator suite under a 60X oil-immersion lens. To avert experimenter selection bias all size measurements were performed on profiles marked during the systematic sampling for counts (average of 30 markers per subject/region/measurement).

### Statistical analysis

Comparisons of AT8 pathology across CTE stages were assessed using the Kruskal-Wallis (KW) with Dunn post-hoc and a Bonferroni correction (KWDB) (corrected *p* < 0.025, two tail, calculated in R using library dunn test) [27, 53]. The Mann Whitney U (MWU) test for significance (corrected *p* < 0.02) was used to compare early (II) and late (III and IV) stages, as described previously [66]. The TauC3 subsample was assessed across stages using the MWU, since the KWDB with chi-square distribution is too stringent for the subsampling (determined using subsample bootstrapping from the AT8 group). Number of TauC3-positive neurons, neurite clusters, and dystrophic fibers was compared to those immunolabeled for AT8 using the Friedman test (FT), which relies on a chi square distribution and provides a conservative assessment of within subject patterns. Associations were assessed using the Spearman rank-order correlation for tied ranks with the Benjamini-Hochberg false discovery method to derive critical values (SRBH, corrected *p* < 0.021) [11]. To determine whether tau pathology follows a regional progression across CTE stages, AT8 counts were plotted and a linear model was used to fit orthogonal polynomials with degrees two through five, using noniterative communication in the MTL as a basis for the ordinate. We used the Akaike Information Criterion (AIC) to determine which model best fit the data [14, 68]. Statistical analyses were run in R (3.3.2, 2016 release “Sincere Pumpkin Patch”) and using custom-designed (CMK) Excel spreadsheets (2013, Microsoft Office, Redmond, WA, USA) based on established statistical formulae. Alpha level for significance was initially set at 0.05, two-tailed, with corrected *p* values as listed.

## Results

### Cohort demographics

Table 1 shows the demographics of the 40 male CTE cases examined with an age range of 24–86 years at time of death. Fourteen cases were stage II, of which 12 played American football (6 professional and 5 college), 13 were stage III, of which 12 played American football (10 professional and 2 college), and 13 were stage IV, all of which played American football (8 professional and 5 college) (Fig. 1A). One stage II, and two stage IV athletes were also military veterans (Table 1). Table 2 shows the history of symptom onset in relation to athletic career, and Table 3 shows apolipoprotein E (ApoE) genotype, Braak stage and Thal phase, where available.

**Table 2:** Symptom onset data from contact sport athletes

Stage	n	Age at symptom onset^a^	Retirement to symptom onset (years)	Symptom onset to death (years)
II	11^b^	36 ± 16 (17–62)	10 ± 16 (− 10–36)	12 ± 10 (1–34)
III	13	44 ± 15 (27–70)	17 ± 17 (− 6–47)	21 ± 12 (5–41)
IV	13	57 ± 14 (29–81)	29 ± 17 (2–54)	17 ± 11 (4–37)

^a^numbers presented as mean ± SD (range)

^b^two CTE stage II individuals had no reported symptoms, and one CTE stage II individual had an unknown age of retirement

**Table 3:** Braak, Thal, and ApoE status of forty contact sport athletes with post mortem diagnosis of CTE

CTE stage	ApoE ε/ε ^a^	% ^b^	Braak stages	%	Thal phases	%
II	0 ε2/ε3	(0)	6 0	(55)	9 0	(82)
	9 ε3/ε3	(82)	4 I-II	(36)	2 1–3	(18)
	2 ε3/ε4	(18)	1 III-IV	(9)	0 4–5	(0)
	3 na^c^		0 V-VI	(0)	3 na	
			3 na			
III	1 ε2/ε3	(25)	2 0	(15)	7 0	(54)
	2 ε3/ε3	(50)	2 I-II	(15)	4 1–3	(31)
	1 ε3/ε4	(25)	7 III-IV	(54)	2 4–5	(15)
	9 na		2 V-VI	(15)	0 na	
			0 na			
IV	0 ε2/ε3	(0)	0 0	(0)	0 0	(0)
	6 ε3/ε3	(60)	0 I-II	(0)	5 1–3	(56)
	4 ε3/ε4	(40)	9 III-IV	(100)	4 4–5	(44)
	3 na		0 V-VI	(0)	4 na	
			4 na			

^a^apolipoprotein E allele genotype

^b^percentage of cases with data

^c^not available

To determine factors associated with AT8 pathology, we analyzed the distribution of subjects across CTE stage in relation to case demographics. We found stage II brains were from younger athletes (mean age at death 48 years) than stage IV (mean age at death 74 years) (*p* < 0.001, KWDB; Table 1, Fig. 1B). Correspondingly, there was a shorter interval between retirement and death in stage II (mean interval of 23 years) compared to stage IV (mean interval of 46 years) athletes (*p* < 0.01, KWDB; Table 1, Fig. 1B). Age at symptom onset was younger (*p* < 0.01, KWDB) and interval between retirement and symptom onset (when present) was shorter (*p* = 0.0201, KWDB) in stage II (36 years and 10 years, respectively) compared to stage IV (57 years and 29 years, respectively) (Table 2, Fig. 1B). There were no differences across CTE stage for age an athlete started playing contact sports (overall average of 12 years), the number of years played (overall average of 15 years), or the age at retirement (overall average of 27 years) (Table 1, Fig. 1B). Similarly, there was no difference across CTE stage for interval from symptom onset to age at death (overall average of 17 years; Table 2, Fig. 1B).

### MTL tau pathology

**Fig. 4:**
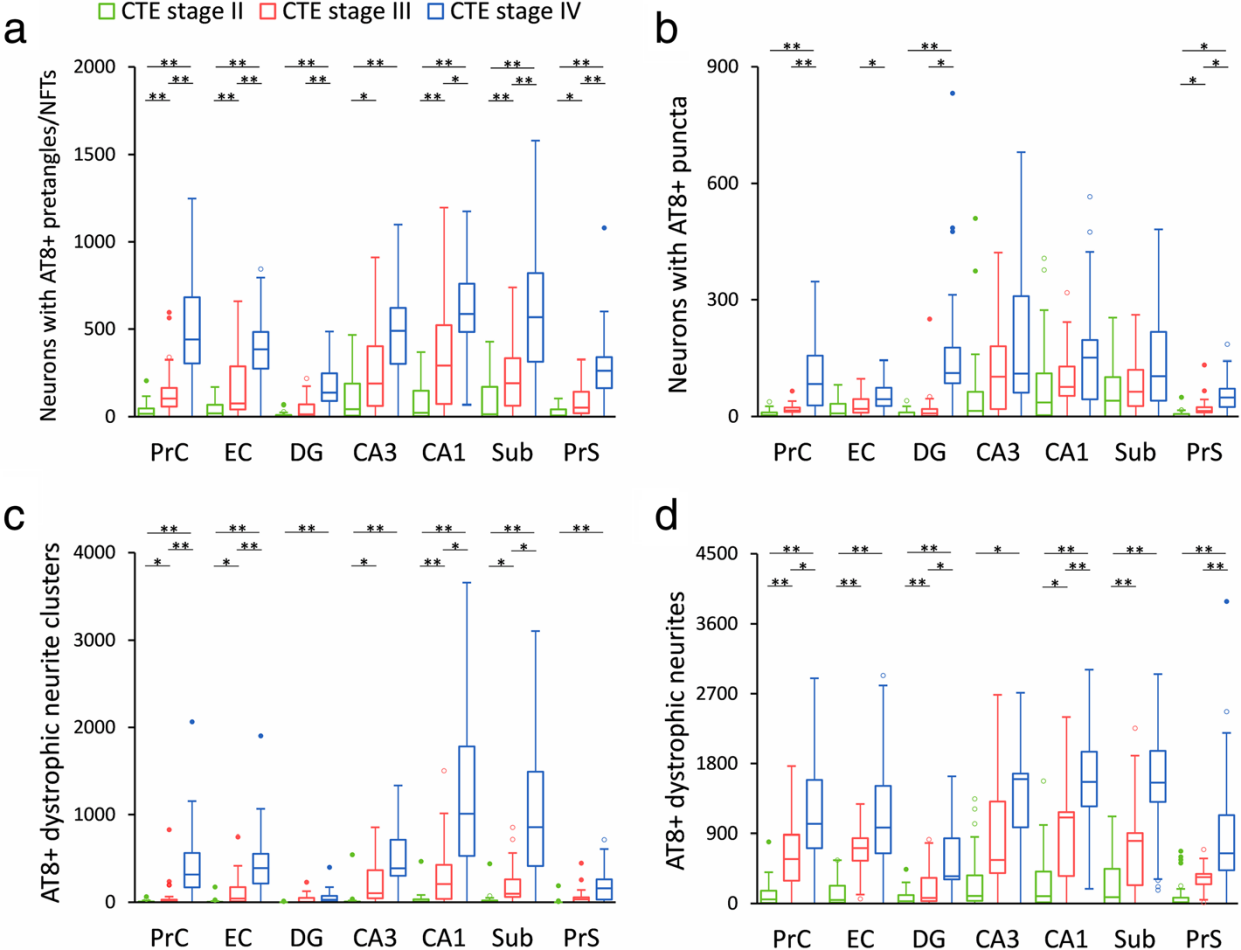
Quantitation of AT8-positive neurons in the MTL across CTE stages II, III, and IV. **a**-**d** Box plots show results from stereological quantitative measures of AT8-positive neurons with NFT (**a**) or puncta (**b**), as well as neurite clusters (**c**) and dystrophic neurites (**d**) across CTE stage II (green), stage III (red), and stage IV (blue). Statistical significance was determined using Kruskal-Wallis, Dunn post-hoc with a Bonferroni correction. * *p* < 0.05, ** *p* < 0.01

Analysis revealed that the MTL displayed four distinct AT8 positive profiles: (1) AT8-positive neurons with intense confluent labeling (Fig. 3b, c); (2) AT8-positive neurons displaying tau puncta (Fig. 3a, b); (3) AT8-positive neurite clusters consisting of aggregates of swollen neurites lacking a discernable soma (Fig. 3d–f); (4) AT8-positive dystrophic swollen neurites (Fig. 3g). TauC3 immunolabeling consisted of tangle-bearing neurons (Fig. 3i-k) and dystrophic neurites. Minimal TauC3-positive dystrophic neurite clusters were observed and neurons with containing tau puncta were difficult to discern, resulting in no evaluation of the latter. Lastly, CA3 hippocampal dendrites displayed AT8-positive thorny excrescences, independent of CTE stage (Fig. 3g, h), similar in structure to that previously reported [13, 29, 36, 54].

### Quantitation of MTL AT8-positive tau profiles

The overall number of AT8-positive profiles differed across CTE stages: stage II displayed the lowest, stage IV the highest, and stage III an intermediate number of total combined AT8-positive lesions within the MTL (Fig. 2b-d). Interestingly, there was variability within each stage, and overlap between stages II and III and stages III and IV (Fig. 2c). Quantitative analysis revealed stage II had less AT8-positive tau pathology in all regions examined compared to stages III and IV (*p* < 0.005, MWU). There was a significant increase in CA1 AT8 positive neuron numbers and neurite clusters in stage III compared to stage II by an average 5.7-fold (*p* < 0.02, KWDB; Fig. 4a, c). CA3 neurite clusters were increased by 4.7-fold in stage III compared with stage II (*p* < 0.02, KWDB; Fig. 4c). AT8-positive dystrophic neurites were significantly increased (by an average 7.2-fold) in PrC, EC, and Sub in stage III compared to stage II (*p* < 0.005, KWDB; Fig. 4d).

Quantitative analysis revealed significantly more AT8-positive tau profiles in all MTL regions in stage IV compared with stage II (Fig. 2c), which included a 9.0-fold increase in AT8-positive neurons (*p* < 0.001, KWDB, Fig. 4a), a 26.7-fold increase in neurite clusters (*p* < 0.01, KWDB, Fig. 4c) and a 6.5-fold increase in dystrophic neurites (*p* < 0.001, KWDB, Fig. 4d). Significant increases in number of neurons displaying AT8 positive puncta were found in stage IV compared with stage II in the PrC, EC, DG, and PrS (by 11.2-fold, *p* < 0.01, KWDB, Fig. 4b).

Although there was an overall 1.5-fold increase in number of tau profiles in stage IV compared with stage III (Fig. 2), this varied by lesion type across MTL regions. CA1 AT8-positive dystrophic neurites were significantly more numerous in stage IV compared to stage III (by 1.0-fold, *p* < 0.025, KWDB, Fig. 4d), while no significant difference in number of AT8-positive neurons (Fig. 4a), or neurite clusters (Fig. 4c) was found in CA1. By contrast, stage IV had significantly more AT8-positive neurons in PrC, EC, DG, Sub, and PrS (by 2.0-fold, *p* < 0.025; KWDB), neurite clusters in PrC and EC (by 3.4-fold, *p* < 0.025, KWDB; Fig. 4c), and dystrophic neurites in PrS (by 4.0-fold, *p* < 0.01, KWDB, Fig. 4d) compared to stage III. Combining all tau profiles revealed a significant increase in AT8-positive tau burden in stage IV compared to stage III in the DG, CA1, and PrS (by 2.1-fold, *p* < 0.025, KWDB).

### TauC3-positive profiles

Stereological quantitative assessment of TauC3-positive lesions revealed significantly higher numbers in stage IV compared with stage II for neurons (*p* < 0.03, MWU), neurite clusters (*p* < 0.03, MWU), and dystrophic neurites (*p* < 0.03, MWU) in all regions but the DG (*p* = 0.05, MWU). CTE stage III had significantly more TauC3-positive neurons (*p* < 0.01, MWU), neurite clusters (*p* < 0.01, MWU), and dystrophic neurites than stage II in the PrC (*p* < 0.01, MWU), and significantly less than stage IV in the PrC (*p* < 0.03 neurons, *p* < 0.03 neurite clusters, *p* < 0.03 dystrophic neurites, MWU) and PrS (*p* < 0.02 neurons, *p* < 0.02 neurite clusters, *p* < 0.02 dystrophic neurites, MWU). When AT8 and TauC3 values were compared there was a strong within-subject correlation between the number of AT8-positive and TauC3-positive profiles (r_s_ = 0.85, *p* < 0.01). Similarly, the distribution of TauC3 pathology across the MTL was similar to that seen with AT8. Within subject comparison confirmed that AT8 pathology was consistently higher than TauC3 pathology for all regions, independent of CTE stage (*p* < 0.05, FT).

### MTL neuron size across CTE stages

Analysis revealed a trend for smaller AT8 positive neurons associated with higher CTE stage throughout the EC. AT8-positive EC neurons were 28.8% larger in stage II (247.6 μm^2^) compared to stage III (181.3 μm^2^; *p* = 0.06, MWU) and 26.2% larger in stage II compared with stage IV (208.9 μm^2^, *p* = 0.08, MWU). There was no difference between stages III and IV in AT8-positive neuron size in any region examined (*p* > 0.50, MWU).

### Neurite cluster and dystrophic neurite size across CTE stage

AT8-positive neurite cluster size was 68.2% larger in PrS of stage IV compared to stage II (*p* = 0.06, KWDB) and 20.0% larger in CA1 of stage III compared to stage IV (*p* = 0.07, KWDB). AT8-positive dystrophic neurite thickness was greater in PrC (16.1%, *p* = 0.04, KWDB), CA1 (20.2%, *p* = 0.04, KWDB), Sub (16.9%, *p* = 0.03, KWDB), and PrS (18.9%, *p* = 0.06, KWDB) of stage II compared to stage III. In contrast, the only increase in AT8-positive neurite thickness was seen in PrC of stage IV compared to stage II (15.4%, *p* = 0.05, KWDB).

### Presence of neurite clusters differentiate early and late CTE stage

Quantitative analysis revealed that for every one neurite cluster observed in stage II, stages III and IV averaged 19 in PrC (*p* < 0.005, MWU), 12 in EC (*p* < 0.001, MWU), 26 in DG (*p* < 0.025, MWU), 7 in CA3 (*p* < 0.005, MWU), 12 in CA1 (*p* < 0.001, MWU), 9 in Sub (*p* < 0.001, MWU), and 7 in PrS (*p* < 0.005, MWU; Fig. 4c). Notably, this type of tau lesion was frequently absent in stage II (Table 4) suggesting less severe axonal/dendritic degeneration early in the disease.

**Table 4:** Number of cases without AT8 tau pathology

	Neurons with NFT	Neurons with punctate p-tau	Total neurons^a^	Neurite clusters	Dystrophic neurites	Total pathology^b^
PrC^c^	4 / 1 / 0^d^	6 / 2 / 1	4 / 0 / 0	9 / 3 / 0	0 / 0 / 0	0 / 0 / 0
EC	6 / 0 / 0	6 / 2 / 0	5 / 0 / 0	9 / 2 / 0	2 / 0 / 1	2 / 0 / 0
DG	7 / 3 / 0	7 / 3 / 2	5 / 2 / 0	9 / 4 / 3	4 / 0 / 0	4 / 0 / 0
CA3	4 / 0 / 0	5 / 3 / 1	3 / 0 / 0	10 / 2 / 0	2 / 0 / 0	2 / 0 / 0
CA1	3 / 0 / 0	3 / 0 / 2	1 / 0 / 0	6 / 0 / 0	1 / 0 / 0	0 / 0 / 0
Sub	5 / 0 / 0	5 / 1 / 2	5 / 0 / 0	9 / 3 / 2	5 / 0 / 0	4 / 0 / 0
PrS	6 / 1 / 0	10 / 2 / 1	5 / 0 / 0	11 / 5 / 0	4 / 0 / 0	3 / 0 / 0

^a^NFT-neurons and punctate p-tau neurons

^b^NFT-neurons, punctate p-tau neurons, neurite clusters, dystrophic neurites

^c^*PrC* Perirhinal cortex, *EC* Entorhinal cortex, *DG* Dentate gyrus, *CA3* Hippocampus CA3, *CA1* Hippocampus CA1, *Sub* Subiculum, *PrS* Pre−/parasubiculum

^d^number of cases with no pathology, presented as CTE stage II/III/IV

### Hippocampal CA subfields and subiculum display the highest tau pathology across CTE stage

**Fig. 5:**
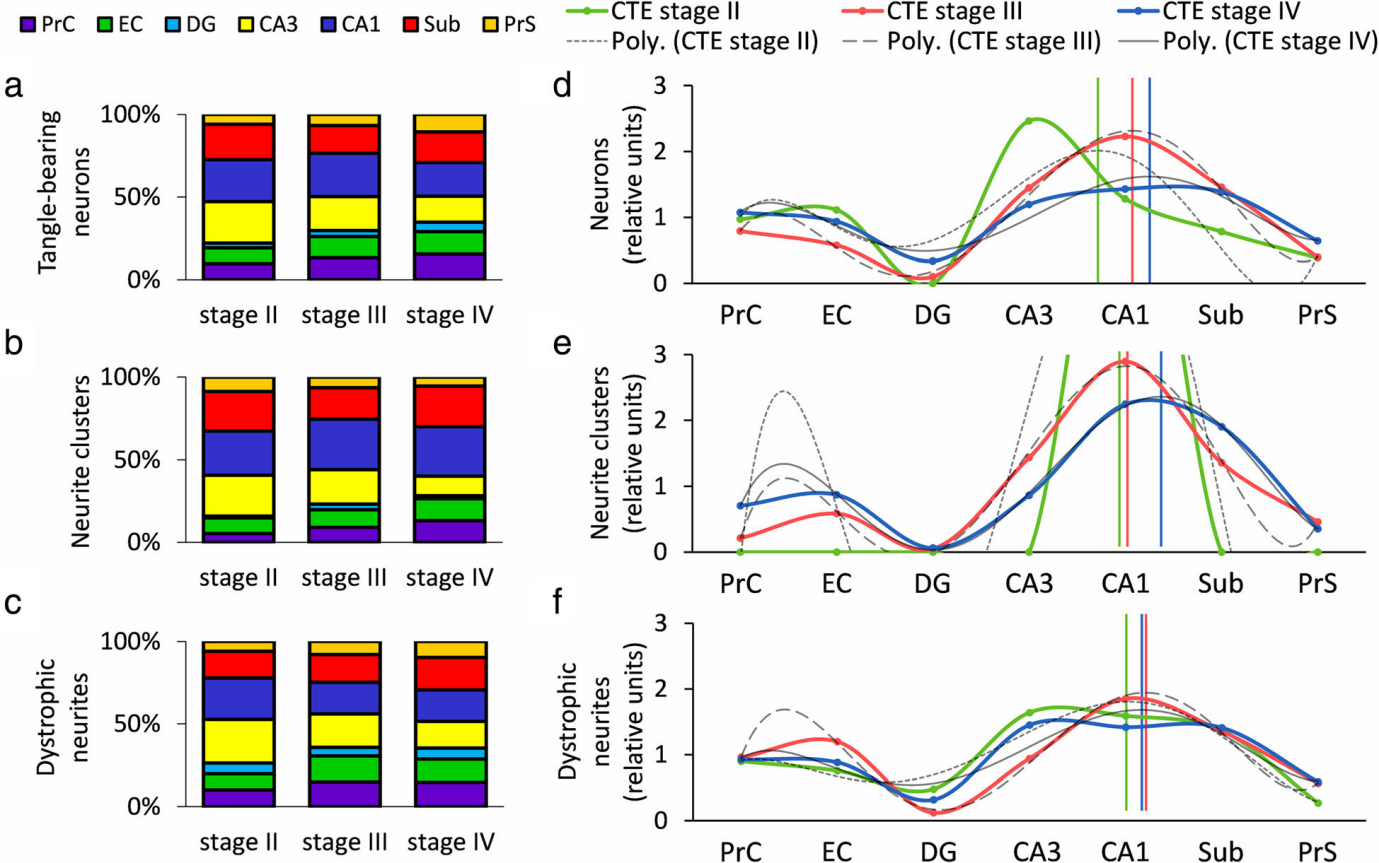
Stacked ratio bar graphs and smoothed line graphs show CA1, CA3, and Sub to be the most severely affected regardless of CTE stage. **a**–**c** Hippocampal CA1 and CA3 as well as the subiculum (Sub) displayed the highest degree of AT8-positive tau pathology at all stages for number of neurons (**a**), neurite clusters (**b**), and dystrophic neurites (**c**). (Purple, PrC, perirhinal cortex; green, EC, entorhinal cortex; aqua, DG, dentate gyrus; yellow, CA3; blue, CA1; red, Sub; orange, PrS, pre−/parasubiculum) **d**–**f** Apex of pathology centered around CA1, which has afferents from CA3 and efferents to the Sub. Vertical lines represent apex of a standardized polynomial-fit regression (degree 5), applied for each stage. AT8-positive pathological tau lesions showed similar patterns for number of neurons (**d**), neurite clusters (**e**), and dystrophic neurites (**f**)

A within-group comparison of regional pathology across all stages revealed that CA3, CA1, and Sub contained the highest number of tau lesions (a combined 55–75% of total, Fig. 5a-c). DG was the least affected region, accounting for an average 4% of AT8-positive pathology (Fig. 5a-c). AT8-positive tau lesion load (defined as the combined number of AT8-positive neurons, neurite clusters, and dystrophic neurites) was plotted based on the concept of directional connectivity within the MTL memory circuit and collapsed by curve-fitting. AIC determined that the best model was a 5-degree logarithmic curve fitted to tau lesion distribution. Examination of the fitted asymptote revealed that CA1 was the most severely affected MTL region between CTE stages (neurons: stage II, *R*^2^ = 0.53, stage III, *R*^2^ = 0.99, stage IV, *R*^2^ = 0.89; neurite clusters: stage II, *R*^2^ = 0.72, stage III, *R*^2^ = 1.00, stage IV, *R*^2^ = 1.00; dystrophic neurites: stage II, *R*^2^ = 0.89, stage III, *R*^2^ = 0.99, stage IV, *R*^2^ = 0.79; Fig. 5).

### Correlation of tau pathology with age and interval between retirement and death

**Fig. 6:**
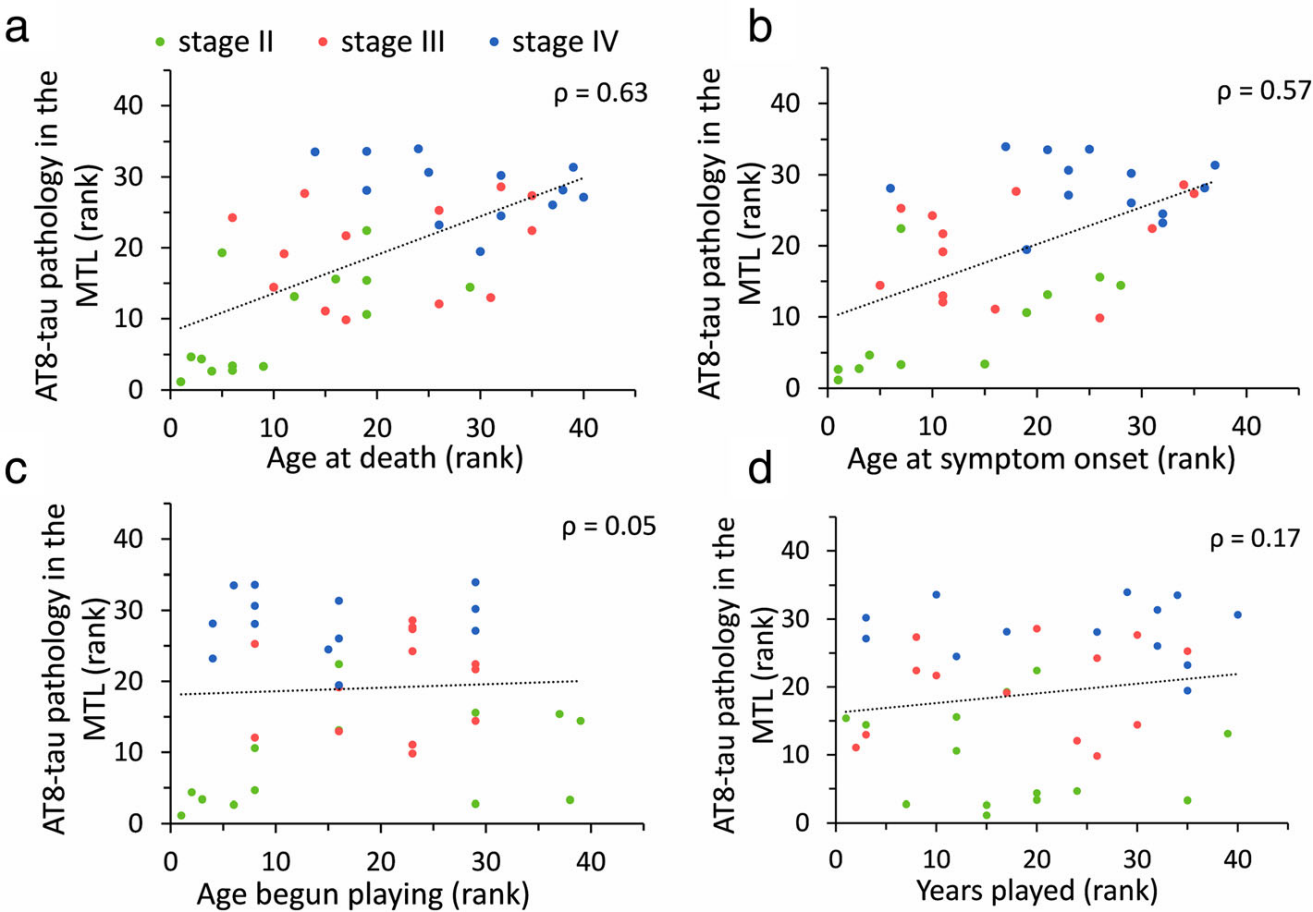
Correlation plots demonstrate a strong association for degree of tau pathology with age at death (**b**) and age at symptom onset (**c**). In contrast, there is no association between number of years started playing (**c**) and overall tau pathology, (**d**) defined as AT8-positive neurons, dystrophic neurites, and neurite clusters, averaged across all MTL regions. ρ, Spearman rank coefficient

Combined across all CTE stages, the number of AT8-positive neurons, neurite clusters and dystrophic neurites correlated positively with age at death for all regions examined (Table 5, Fig. 6a). Combining all stages revealed a significant positive correlation for interval between retirement and death (mean = 35.4 years, SD = 17.4 years; Table 1) with number of AT8 bearing neurons (average r_s_ = 0.51, Table 5), neurite clusters (average r_s_ = 0.50, Table 5), and dystrophic neurite number for all regions examined (average r_s_ = 0.45, Table 5). Results from age-adjusted regression models demonstrated that CTE stage revealed the strongest association with neurite clusters across all regions except hippocampal subfield CA3 (Table 6).

**Table 5:** Association between athlete age and AT8 p-tau pathology

	Neurons with NFT	Neurons with punctate p-tau	Total neurons^a^	Neurite clusters	Dystrophic neurites	Total pathology^b^
Age at death						
PrC^c^	0.64^d^ ***	0.55 ***	0.65 ***	0.66 ***	0.55 ***	0.63 ***
EC	0.65 ***	0.49 **	0.61 ***	0.63 ***	0.49 **	0.60 ***
DG	0.60 ***	0.63 ***	0.67 ***	0.51 **	0.63 ***	0.66 ***
CA3	0.57 ***	0.18	0.48 **	0.59 ***	0.50 **	0.53 ***
CA1	0.58 ***	0.22	0.48 **	0.65 ***	0.53 ***	0.61 ***
Sub	0.61 ***	0.27	0.56 ***	0.49 **	0.46 **	0.57 ***
PrS	0.58 ***	0.57 ***	0.62 ***	0.49 **	0.53 ***	0.59 ***
Years from retirement to death						
PrC	0.52 ***	0.40 *	0.52 ***	0.52 ***	0.41 **	0.49 **
EC	0.53 ***	0.49 **	0.50 **	0.54 ***	0.38 *	0.50 **
DG	0.42 *	0.62 ***	0.54 **	0.45 **	0.54 **	0.56 ***
CA3	0.48 **	0.15	0.38 *	0.45 **	0.37	0.40 *
CA1	0.46 **	0.19	0.38 *	0.58 ***	0.46 **	0.53 ***
Sub	0.60 ***	0.30	0.56 ***	0.52 ***	0.45 **	0.56 ***
PrS	0.52 ***	0.47 **	0.54 ***	0.43 **	0.54 ***	0.57 ***

^a^Neurons with NFT and punctate p-tau neurons

^b^Neurons with NFT, punctate p-tau neurons, neurite clusters, dystrophic neurites

^c^*PrC* Perirhinal cortex, *EC* Entorhinal cortex, *DG* Dentate gyrus, *CA3* Hippocampus CA3, *CA1* Hippocampus CA1, *Sub* Subiculum, *PrS* Pre−/parasubiculum

^d^Spearman rank order correlation with Benjamini-Hochberg false discovery critical values, * *p* < 0.05, ** *p* < 0.01, *** *p* < 0.001

**Table 6:** Regional age-adjusted regression estimates of CTE tau pathology

	Neurons with NFT	Neurons with punctate p-tau	Neurite clusters	Dystrophic neurites
PrC^a^	0.95 (0.46, 1.44)^b^ ***	0.89 (0.37, 1.41) ***	1.59 (0.94, 2.23) ***	0.88 (0.44, 1.31) ***
EC	0.99 (0.44, 1.54) **	0.41 (−0.15, 0.95)	1.50 (0.75, 2.23) ***	0.95 (0.93, 0.98) ***
DG	0.77 (−0.10, 1.58)	1.10 (0.13, 2.05) *	0.61 (0.50, 0.71) ***	0.63 (−0.09, 1.34)
CA3	0.50 (−0.07, 1.06)	0.19 (−0.62, 0.98)	0.76 (− 0.20, 1.66)	0.44 (− 0.12, 0.99)
CA1	0.67 (0.12, 1.20) *	0.26 (−0.34, 0.85)	1.22 (0.55, 1.86) ***	0.55 (0.05, 1.03) *
Sub	0.69 (0.14, 1.22) *	0.32 (0.27, 0.38) ***	1.29 (0.36, 2.17) *	1.06 (0.34, 1.76) **
PrS	1.10 (0.55, 1.64) ***	0.80 (0.16, 1.43) *	1.12 (0.21, 2.01) *	0.79 (0.26, 1.30) *

^a^*PrC* Perirhinal cortex, *EC* Entorhinal cortex, *DG* Dentate gyrus, *CA3* Hippocampus CA3, *CA1* Hippocampus CA1, *Sub* Subiculum, *PrS* Pre−/parasubiculum

^b^age-at-death associated regression coefficient (95% confidence interval) with false discovery rate α = 0.03, * *p* < 0.05, ** *p* < 0.01, *** *p* < 0.001

### Correlation between years played and severity of tau pathology

Length of athletic career, which ranged from 5 to 30 years (mean = 15.4 years, SD = 6.1 years; Table 1), did not correlate with CTE stage (χ^2^ (2, 40) = 2.335, *p* = 0.311, KW), or severity of tau pathology (AT8-positive neurons, neuritie clusters, or dystrophic neurites) in any region examined (Fig. 6d). Similarly, age at which the athlete began playing contact sports (range 5 to 18 years of age, mean = 11.7 years, SD = 2.8 years; Table 1) was not associated with CTE stage (χ^2^ (2, 40) = 2.658, *p* = 0.265, KW) or number of AT8-positive lesions (neurons, neuritie clusters, or dystrophic neurites) in any region examined (Fig. 6c).

### Tau pathology association with age at retirement

Age at retirement (range 18 to 42 years, mean = 27.2 years, SD = 6.2 years; Table 1) positively correlated with AT8-positive neurons in the DG (r_s_ = 0.43, *p* < 0.05, SRBH) and CA1 (r_s_ = 0.39, *p* < 0.05, SRBH). Additionally, a significant positive correlation was observed between age at retirement and AT8-positive dystrophic fibers in CA3 (r_s_ = 0.40, *p* < 0.05, SRBH). Number of neurite clusters did not correlate with age at retirement for any MTL region evaluated. There was no association between age at retirement and AT8-positive neurons or dystrophic neurites in PrC, EC, Sub, or PrS.

### Tau pathology correlates with older age of symptom onset

In each MTL region examined, more severe tau pathology was associated with later age of symptom onset (mean = 46.6 years, SD = 17.1 years; Table 7, Fig. 6b). Severity of tau pathology (number of AT8-positive neurons, neurite clusters, and dystrophic neurites) positively correlated with longer interval between retirement and symptom onset (average r_s_ = 0.42, Table 7). Years between symptom onset and death were not significantly correlated with tau pathology in any MTL region examined.

**Table 7:** Association between symptom onset and AT8 tau pathology

	Neurons with NFT	Neurons with punctate p-tau	Total neurons^a^	Neurite clusters	Dystrophic neurites	Total pathology^b^
Age at symptom onset						
PrC^c^	0.59^d^ ***	0.46 **	0.58 ***	0.65 ***	0.46 **	0.53 ***
EC	0.62 ***	0.57 ***	0.57 ***	0.50 **	0.29	0.47 **
DG	0.63 ***	0.41 *	0.58 ***	0.41 *	0.61 ***	0.60 ***
CA3	0.47 **	0.16	0.43 *	0.42 *	0.51 **	0.48 **
CA1	0.50 **	0.23	0.43 **	0.58 ***	0.50 **	0.59 ***
Sub	0.52 ***	0.26	0.48 **	0.41 *	0.41 *	0.51 **
PrS	0.61 ***	0.52 ***	0.65 ***	0.49 **	0.50 **	0.57 ***
Years from retirement to symptom onset						
PrC	0.50 **	0.36	0.49 **	0.57 ***	0.38 *	0.45 **
EC	0.52 **	0.53 **	0.48 **	0.44 **	0.22	0.40 *
DG	0.50 **	0.41	0.49 **	0.39	0.51 **	0.50 **
CA3	0.40 *	0.09	0.33	0.34	0.40 *	0.37
CA1	0.41 *	0.17	0.33	0.54 ***	0.45 **	0.53 ***
Sub	0.52 **	0.24	0.48 **	0.44 **	0.42 *	0.52 ***
PrS	0.56 ***	0.49 **	0.58 ***	0.45 **	0.50 **	0.55 ***

^a^Neurons with NFT and punctate p-tau neurons

^b^Neurons with NFT, punctate p-tau neurons, neurite clusters, dystrophic neurites

^c^*PrC* Perirhinal cortex, *EC* Entorhinal cortex, *DG* Dentate gyrus, *CA3* Hippocampus CA3, *CA1* Hippocampus CA1, *Sub* Subiculum, *PrS* Pre−/parasubiculum

^d^Spearman rank order correlation with Benjamini-Hochberg false discovery critical values, * *p* < 0.05, ** *p* < 0.01, *** *p* < 0.001

### CTE and Aβ pathology

**Fig. 7:**
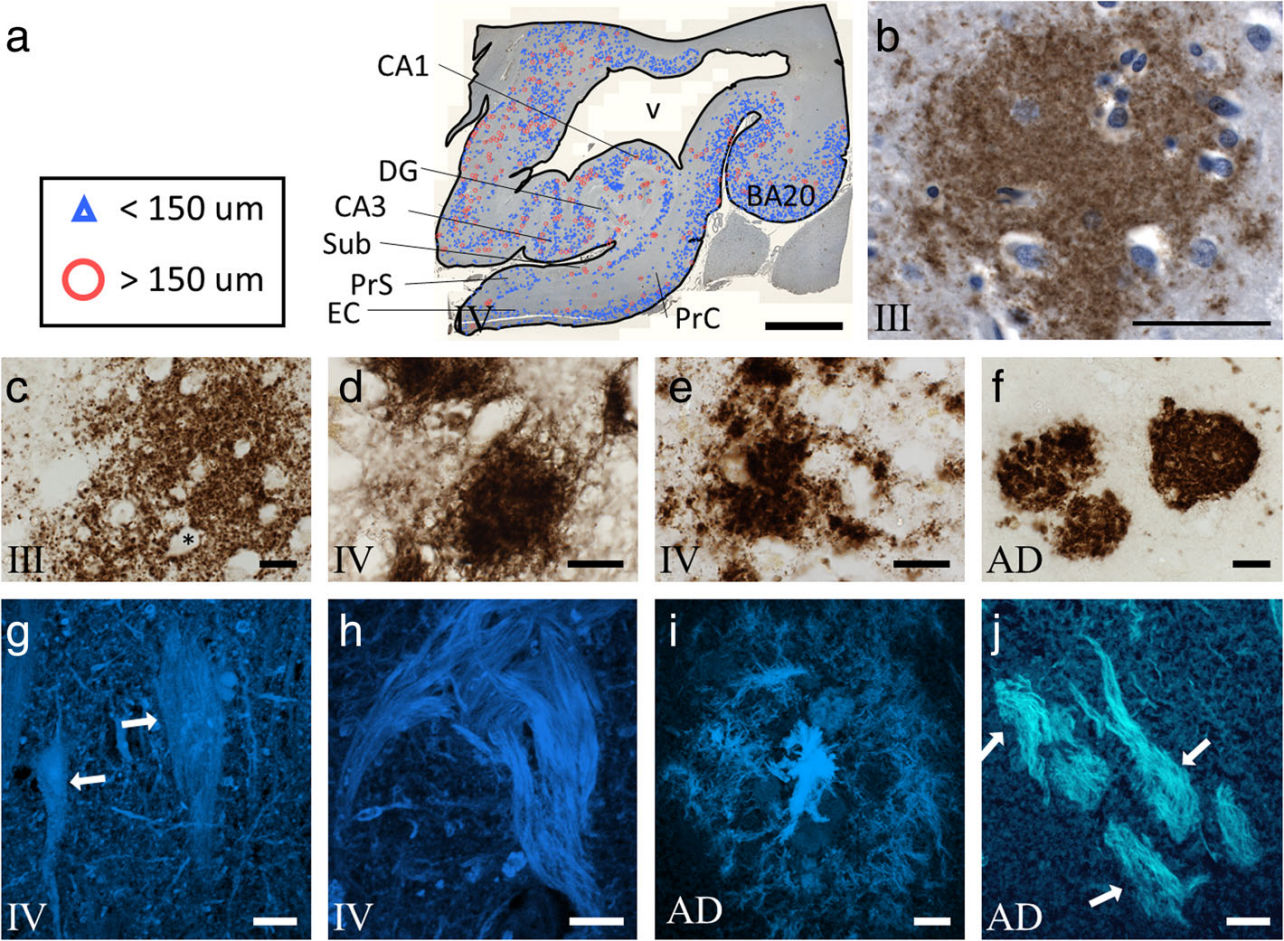
Photomicrographs illustrate Aβ pathology in the MTL of CTE. **a** Charting of the distribution of 6E10 positive diffuse amyloid plaques (blue and red) superimposed upon the original immunolabeled section from a CTE stage IV case. **b** MTL sections immunolabeled with 6E10 (brown) and Nissl counter stained (blue) show a diffuse plaque (**b**) in the inferior temporal cortex (BA20) of a stage III case. **c**-**f** Photomicrographs of Aβ_1–42_ immunolabeling in the PrS of CTE stage III (**c**), EC (**d**) and PrS (**e**) of stage IV, and CA3 from an AD case (**f**). Note the large amorphous shape of a diffuse plaque fenestrated with blood vessels (black asterisk) in stage III (**c**) compared to more compact appearing plaques in stage IV (**d** and **e**). In contrast, Aβ_1–42_ plaques appeared as dense compact spheroids in AD (**f**). **g**-**j** Thioflavine S positive NFTs (white arrows) in CA1 (**g**) and EC (**h**) in a CTE stage IV case. Note the difference between the smooth and flowing string-like NFTs in CTE (**g** and **h**), contrasted with flame-like NFTs found in AD (**j**). Thioflavine S staining revealed dense core plaques with fibril pathology in the CA1 subfield in AD (**i**) but not in CTE. Abbreviations: AD, Alzheimer’s disease; DG, dentate gyrus; EC, entorhinal cortex; PrC, perirhinal cortex; PrS, presubiculum; Sub, subiculum; v, ventricle. Scale bars = 5000 μm in (**a**), 50 μm in (**b**), 10 μm in (**d**-**f**), and 20 μm in (**g**-**j**)

In contrast to the extensive deposition of tau pathology seen in the MTL, presence of diffuse APP/Aβ (6E10) positive plaques (Fig. 7) was not associated with CTE stage, MTL region, or severity of pathological tau (Additional file 1: Figure S1). To further evaluate APP/Aβ burden, we categorized 6E10-positive plaques by quantity, as 0–1, 2–10, 11–50, or > 50, within three areas: (1) DG and hippocampal subfields CA3 and CA1, (2) Sub and PrS, and (3) EC and PrC. For DG/CA3/CA1, the majority of CTE stage II (71%), stage III (62%), and stage IV (50%) cases had between 11 and 50 6E10-positive plaques. This pathology was less in Sub/PrS and EC/PrC, where the majority of cases had between 2 and 10 6E10-positive plaques in CTE stage II (69 and 69%), stage III (77 and 62%), and stage IV (54 and 50%). In cases with the highest Aβ pathology, 18% of stage II, 15% of stage III, and 43% of stage IV brains had > 50 6E10 positive plaques in CA1/CA3/DG (Fig. 7a, b). An average of 25% of brains had between 11 and 50 6E10-positive plaques in the Sub/PrS and EC/PrC. The distribution of 6E10 pathology did not mimic that seen for AT8 or TauC3. Thioflavine S histochemistry confirmed a lack of neuritic pathology surrounding diffuse 6E10-positive plaques. While we observed thioflavine S-positive NFTs (Fig. 7g, h), neuritic or fibril AD-like plaque pathology was not seen in CTE cases (Fig. 7g-j).

To more closely characterize plaque pathology in CTE, we immunolabeled MTL sections using an antibody against Aβ_1–42_, a pathological species of Aβ that reveals amyloid pathology in AD. Aβ_1–42_-positive lesions were not seen in any MTL region in stage II and were absent in DG, hippocampal subfields CA3, CA1 and Sub of CTE stages III and IV (Additional file 1: Figure S2). However, Aβ_1–42_-positive diffuse plaques were observed in PrS, EC, and PrC of CTE stage III and IV as amorphous non-cored pathology (Fig. 7c-e). By contrast, Aβ_1–42_-positive plaques appeared dense and confined to discrete ovoid deposits in AD (Fig. 7f).

## Discussion

The present study is the first detailed quantitative analysis of tau pathology in the MTL of CTE brains neuropathologically staged according to McKee and colleagues [61]. We analyzed postmortem tissue containing seven MTL subregions from male contact sport athletes with a mean career length of more than a decade. Across stages II, III, and IV, which display an escalation in tau pathology, we found that more advanced CTE stages were associated with older age at death. Conversely, an association with CTE stage was not seen between number of years the athlete played contact sports, age at which sport began, age at retirement, or time interval from retirement to death. The current findings differ from prior reports that found an association between CTE stage and number of years played or time from retirement to death [18, 65, 95], as well as an association with severity of structural changes and years played or age at first exposure to the sport [89, 93]. The differences between the present findings and those of prior investigations may be related to several variables including age at first exposure, age at retirement, age at death, comorbidities, and statistical analyses involving binary separation of pathologies and/or subject information. These factors require further assessment using a larger cohort of well-characterized CTE cases with consistent and thoroughly documented histories. In the present study, examination of overall severity of tau pathology and subject demographics, independent of CTE stage, revealed an association between degree of MTL tau burden (as measured by AT8-positive neurons, dystrophic neurites, and neuritic clusters) and age at death. However, we also found that the degree of tau burden in the MTL associated with age at symptom onset and time from retirement to symptom onset. For example, the older the athlete when symptoms began and the longer the interval between retirement and symptom onset, the more severe the AT8-positive tau burden. Unlike age-at-death, this association was not related to CTE stage. These findings suggest that characterization of the MTL can aide in developing a more sensitive CTE sub-staging schema to track disease progression.

The role tau pathology plays in the onset of CTE and its relationship to functional deficits remains obscure. There is no consensus on whether post mortem confirmation of CTE tau lesions are a true reflection of rTBI [10, 12, 49], single-hit severe TBI (sTBI) [21, 45, 85], or non-concussive environmental insults [70]. Confounds that may affect an athlete’s CTE etiology include high body mass index and acquired systemic conditions (e.g., diabetes and hypertension) [89], which are general health factors associated with cognitive decline seen in non-CTE age-related dementias such as AD [82]. Although there is a higher incidence of neuropsychiatric disorders in athletes with CTE lesions [66, 96], similar behavioral conditions and brain pathology are seen in subjects without a diagnosis of CTE [8, 12, 37, 104, 108] making it difficult to discern the pathophysiological etiology of these ailments. This conundrum also applies to military veterans with post-traumatic stress disorder that present with neuropsychiatric conditions (e.g., depression) and brain structural abnormalities (e.g., decreased hippocampal volume) similar to CTE [34, 40, 91, 92, 102, 106].

Interestingly, we did not find evidence for connectivity-based propagation of tau pathology across the sectors of the MTL memory circuit. In the healthy adult brain, tau is mainly found in axons as part of the microtubule structure and transport system [46]. However, in various tauopathies, tau undergoes post-translational modifications including phosphorylation, acetylation, methylation, truncation, and resulting conformational alterations [59, 81, 101]. In AD and other tauopathies, aberrant tau dissociates from microtubules, disrupting assembly, steady state, and microtubule-dependent transport [5, 103]. Histologically, this dissociation appears as an accumulation of intraneuronal aggregates of hyperphosphorylated tau and neuritic pathology in the form of swollen neurites and dystrophic neuritic clusters [43, 44, 103]. The greater extent of neuritic pathology across escalating CTE stages found in the current study, suggests that rTBI results in a chronic progressive state of neurite injury involving neuropathological tau modifications, likely with subsequent deficits in axonal transport [94]. In this regard, we found elevated tau neuritic compared to neuronal pathology in CTE, supporting the concept that tau dysregulation has an axonal/dendritic origin that moves towards the soma in a process dependent on retrograde transport of toxic tau, as suggested in AD [59, 101]. Transgenic animal models expressing human tau exhibit propagation of putative toxic species of tau between connectionally interrelated brain regions [20, 26, 48, 57], which may be associated with a prion-like template misfolding [26]. Based upon basic MTL connectional anatomy, propagation of pathological tau would entail progression from the EC to DG, then to CA3, CA1, and Sub [50, 51, 99]. However, we found that the most affected regions were hippocampal CA3, CA1 subfields and Sub, while the least affected were DG and PrS, with an intermediate range represented by EC and PrC. These observations do not lend support to a prion-like propagation of modified tau within the MTL circuit.

We investigated whether any MTL subregion is more susceptible to tau pathology in an early stage of CTE. Previous findings have highlighted that hippocampal CA4 and CA2 subfields are more prone to tau pathology in CTE [7, 61], whereas CA3 is associated with greater pathology in AD [13]. Here, we found that CA1 was the most and DG the least susceptible to tau pathology in CTE. The difference between the current findings and those of others may be related to the types of tau pathology evaluated and differences in the extent of the hippocampal complex examined. For example, although other studies included the entire CA4 field [7, 61], our tissue blocks lacked a complete and well-defined CA4 region. It is important to note that although the DG displayed the least amount of tau pathology, it was not resilient to tau lesions, as evinced by a significant increase in AT8-positive neurons, neurite clusters, and dystrophic fibers in stage IV compared to stage II. These data suggest that tau pathology worsens over time despite cessation of rTBI events in CTE [12, 69], but this needs further investigation. Specifically, in the non-rTBI population, tau pathology appears in 15–30% of individuals in their 20s and 30s and 40–45% of individuals in their 40s, as Braak stage I-II, stages which are far dissociated from clinical presentation of cognitive decline [16, 100]. It is believed that this pathology progresses within an individual. Perhaps part of the advance seen in stage IV compared with stage II associated with age. However, whether rTBI precipitates an onslaught of pathology that is exacerbated by age, or whether tau presents as part of aging, which is exacerbated by pathological processes associated with rTBI is difficult to determine with the current sample size. Our statistical analyses suggest that all MTL areas except CA3 are affected by tau pathology in a stage congruent manner, independent of age. Overall, age is a potential confound that affects tau burden in both AD and CTE.

TauC3 recognizes a form of tau with a conformational change imposed by caspase-3-mediated cleavage at D421, indicative of apoptotic cell death [19, 75]. The current analysis found no differences in TauC3-positive neurons, neurite clusters, or dystrophic neurites in hippocampal subfield CA1 and Sub between CTE stage III and stages II or IV, regions where the number of AT8-positive pathology (neurons, neurite clusters, and dystrophic fibers) significantly differentiated stage III from II and IV. Neurite clusters in PrC were the only MTL TauC3-positive pathology that was different between stage III and stages II or IV. Since in preclinical AD and normal aging, the PrC is one of the earliest regions to show tau pathology, our TauC3 findings may highlight a neuropathological corollary for memory problems observed in CTE [15, 62, 109].

All MTL regions except the DG had significantly more TauC3-positive neurons in stage IV compared with stage II. Previously, we reported a significantly greater percentage of TauC3-positive to total cholinergic basal forebrain (CBF) neurons in CTE stages III or IV compared to stage II [72], indicating a similar pathologic progression across stages between these regions in CTE. By contrast, the percentage of CBF TauC3-positive neurons remained consistent during the progression of AD [101], whereas in the AD MTL, TauC3-positive pathology appears early, progresses with the disease and strongly inversely correlates with cognitive function [30, 38, 83]. Taken together, these findings demonstrate a difference in the onset of and regional development of late stage tau pathology between CTE and AD, while underscoring a possible mechanism for cognitive decline in later CTE stages.

We did not find that APP or Aβ deposition associated with any CTE stage or MTL region. The greatest extent of amyloid-like pathology was observed in inferior temporal cortex (Brodmann area 20), which consisted of diffuse, nonfibrillar plaques positive for APP/AICD/Aβ (6E10). Neither dense core nor neuritic plaque pathology was seen with thioflavine S staining or using an antibody specific for Aβ_1–42_. This Aβ diffuse plaque phenotype is reminiscent of that reported decades after sTBI, which is unrelated to the site of injury [90]. Moreover, we did not find an association between tau and amyloid lesions in any MTL region examined, suggesting that the deposition of Aβ species is not a necessary precondition or co-condition for the onset of tau pathology in CTE. This is in line with other tauopathies, where APP and Aβ deposition does not mirror p-tau deposition and is more likely associated with subject age than degree of cognitive impairment [74, 110]. Of note, the presence and degree of NFTs, along with axonal degeneration, correlate well with cognitive impairment in AD, while Aβ plaque pathology does not [44, 73, 86, 101].

Interestingly, animal studies demonstrate removal or inhibition of toxic tau species in the brain improves performance on a hippocampal-dependent memory task [80, 87], suggesting the potential use of anti-tau drugs for treatment in CTE [80, 84]. Currently, blood biomarkers and MRI volumetrics offer limited promise for determining the presence and progression of CTE-related degeneration [3, 55, 98]. It may be possible to monitor intra-seasonal sport related brain trauma through the evaluation of serum levels of the neurofilament-light chain, but this has not demonstrated good long-term prognostic accuracy to date [76]. Despite the association between tau and mechanical trauma [56], plasma levels of tau are not altered in accord with trauma frequency or severity during the course of an American football season [76]. The current and previous findings [72], suggest different forms of tau pathology provide a diverse set of markers to track CTE development and efficacy of tau-mediated treatments, especially in the pre-symptomatic stages of this disorder [7, 58, 72, 87].

There are limitations to the present study. We examined portions of the MTL from an autopsy cohort containing a heterogeneous population of cases with a history of contact-sport related rTBI. Therefore, those examined may not be representative of the larger CTE population, where concussive and traumatic forces are more variable [35, 88, 105]. Moreover, the tissue we obtained primarily contained the anterior aspect of the MTL and may differ from the location examined in prior studies [65, 95]. Clinical data was obtained retrospectively from family members and may be subject to recall bias. Since we lacked information on position played and practice or game playing time, we were unable to correlate CTE stage and tau pathology with these variables. Prior studies with American football players indicate an increased number and severity of hits for positions along the line of scrimmage and in plays and drills that involve player-player tackling [17, 88]. Future prospective studies should include well documented clinical, psychiatric and athletic history to determine the interaction between subject variables and tau pathologies during the progression of CTE.

## Conclusions

Here we provide evidence that further regional subfield analysis focusing on the MTL memory circuit provides a finer resolution of the progression of p-tau pathology during CTE. In the present study, we observed that the most severe tau pathology appeared in hippocampal CA1, CA3 and Sub. We found a CTE stage-dependent increase in pathology across all MTL regions examined, while not supporting for the concept of circuit-based propagation of tau pathology within the MTL. The current study offers new insight into the extent of tau pathology within the MTL across neuropathological stages of CTE.

## Supplementary information

**Additional file 1: Figure S1.** Low power photomicrographs showing tau and Aβ immunoreactivity in the medial temporal lobe of CTE cases. AT8-immunolabeled sections from a representative CTE stage III (A) and stage IV (C) case contrasted with amyloid (6E10)-immunolabeled sections (B, D) from the same cases. Scale bar = 5000 μm. Abbreviations: CA1 and CA3, hippocampal subfields; DG, dentate gyrus; EC, entorhinal cortex; PrC, perirhinal cortex; PrS, presubiculum; Sub, subiculum. **Figure S2.** Low power photomicrographs showing tau and Aβ immunoreactivity in the medial temporal lobe (MTL) of CTE cases. (A) Aβ_42_ immunoreactive punctate pathology in the inferior temporal cortex (BA20) of a CTE stage III and (B) subicular and entorhinal cortices of a stage IV case. (C) Amyloid (6E10) immunostaining in the MTL. Note the virtual absence of amyloid staining in the MTL of the same case shown in B. Scale bars = 5000 μm. Abbreviations: BA20, inferior temporal cortex; CA1 and CA3, hippocampal subfields; DG, dentate gyrus; EC, entorhinal cortex; PrC, perirhinal cortex; PrS, presubiculum; Sub, subiculum.

## Data Availability

Data collected and analyzed to support the findings made in this publication will be made available upon reasonable request to the corresponding author.
