# Supplementary material for: Tau pathology in the medial temporal lobe of athletes with chronic traumatic encephalopathy: a chronic effects of neurotrauma consortium study
Source: Acta Neuropathol Commun. 2019 Dec 12;7:207. doi: 10.1186/s40478-019-0861-9 (PMC6909582; doi:10.1186/s40478-019-0861-9)
Supplement: Supplementary file 1 — Additional file 1: Figure S1. Low power photomicrographs showing tau and Aβ immunoreactivity in the medial temporal lobe of CTE cases. AT8-immunolabeled sections from a representative CTE stage III (A) and stage IV (C) case contrasted with amyloid (6E10)-immunolabeled sections (B, D) from the same cases. Scale bar = 5000 μm. Abbreviations: CA1 and CA3, hippocampal subfields; DG, dentate gyrus; EC, entorhinal cortex; PrC, perirhinal cortex; PrS, presubiculum; Sub, subiculum. Figure S2. Low power photomicrographs showing tau and Aβ immunoreactivity in the medial temporal lobe (MTL) of CTE cases. (A) Aβ42 immunoreactive punctate pathology in the inferior temporal cortex (BA20) of a CTE stage III and (B) subicular and entorhinal cortices of a stage IV case. (C) Amyloid (6E10) immunostaining in the MTL. Note the virtual absence of amyloid staining in the MTL of the same case shown in B. Scale bars = 5000 μm. Abbreviations: BA20, inferior temporal cortex; CA1 and CA3, hippocampal subfields; DG, dentate gyrus; EC, entorhinal cortex; PrC, perirhinal cortex; PrS, presubiculum; Sub, subiculum. [file 40478_2019_861_MOESM1_ESM.docx]

**Additional file 1**


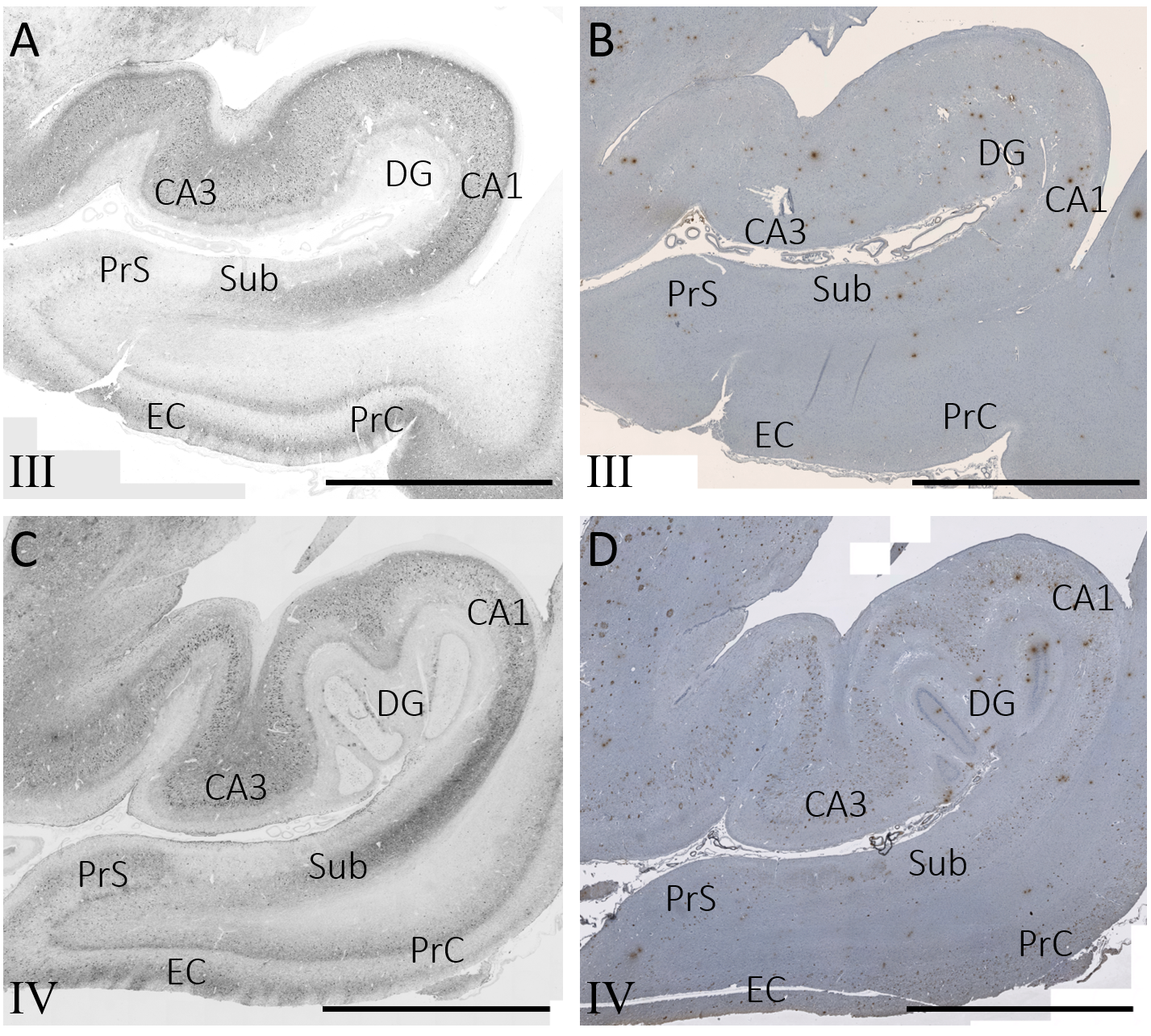


**Figure S1.** Low power photomicrographs showing tau and Aβ immunoreactivity in the medial temporal lobe of CTE cases. AT8-immunolabeled sections from a representative CTE stage III (A) and stage IV (C) case contrasted with amyloid (6E10)-immunolabeled sections (B, D) from the same cases. Scale bar = 5000 µm. Abbreviations: CA1 and CA3, hippocampal subfields; DG, dentate gyrus; EC, entorhinal cortex; PrC, perirhinal cortex; PrS, presubiculum; Sub, subiculum.


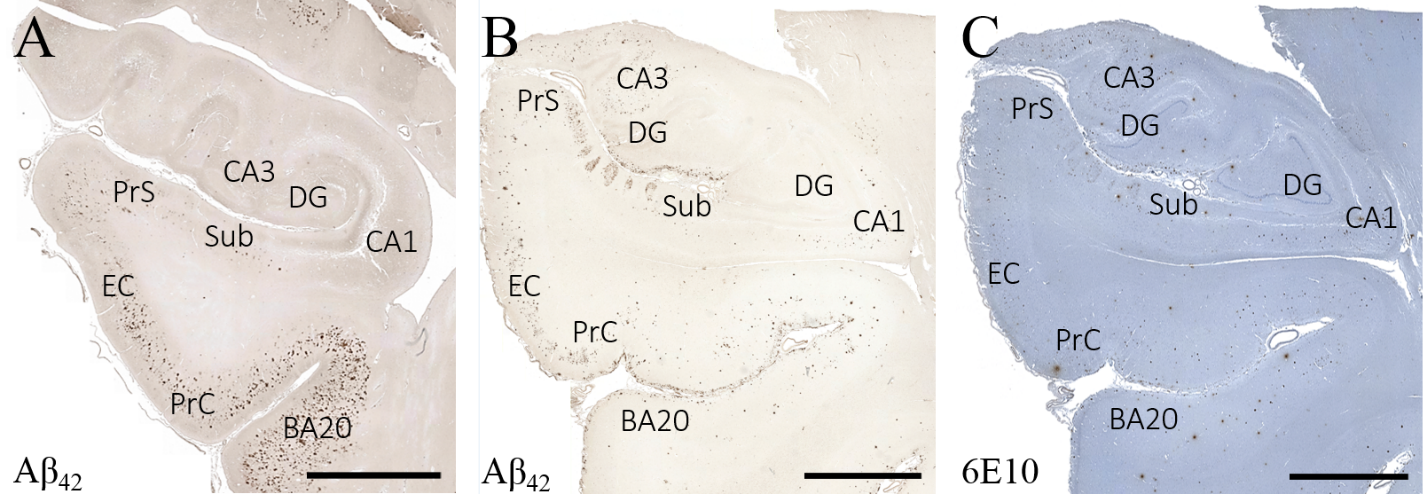


**Figure S2.** Low power photomicrographs showing tau and Aβ immunoreactivity in the medial temporal lobe (MTL) of CTE cases. (A) Aβ_42_ immunoreactive punctate pathology in the inferior temporal cortex (BA20) of a CTE stage III and (B) subicular and entorhinal cortices of a stage IV case. (C) Amyloid (6E10) immunostaining in the MTL. Note the virtual absence of amyloid staining in the MTL of the same case shown in B. Scale bars = 5000 µm. Abbreviations: BA20, inferior temporal cortex; CA1 and CA3, hippocampal subfields; DG, dentate gyrus; EC, entorhinal cortex; PrC, perirhinal cortex; PrS, presubiculum; Sub, subiculum.
